# The Institutional Worker

**Published:** 1912-10-19

**Authors:** 


THE
### INSTITUTIONAL WORKER
**Being a Special Supplement to "The Hospital"**

### Editor's Notices.

**Contributions :**

Contributions should be written, or preferably typed, on one side of the paper only, and all articles sent in are accepted upon the distinct understanding that they are forwarded to The Hospital only.

The Editor cannot undertake to return MSS. not used, but when a stamped directed envelope is enclosed the MSS. may be returned if a special request is made.

Accepted articles and paragraphs of news will be paid for after publication at the scale rate.

**Address :**

To prevent delay all contributions and letters on editorial business must be addressed exclusively to the Editor, "The Hospital," 29 Southampton Street, Strand, London, W.C. It is important that this regulation shall be strictly observed.

**Correspondence :**

Correspondence on all subjects is invited. The name and address of correspondents must be given as a guarantee of good faith, but not necessarily for publication.

**Special Articles :**

Special articles are invited, and questions, inquiries, and paragraphs upon all matters relating to the work, administration, and management of general, special, mental, fever, cottage and convalescent hospitals, sanatoria, homes, institutions, societies and organisations for the treatment or care of the sick, injured and dependants of all classes. Every matter affecting the interests and welfare of the staff of all grades working in these institutions will receive special consideration.

**Administrative Medicine, Research and Items of News :**

Special terms will be paid for approved articles on subjects in this wide field by experts who have made some section of it their special study and interest. We wish to give prominence to every movement tending to advance accuracy of diagnosis, efficiency in treatment, and the development of modern methods to eradicate disease and promote the welfare of the suffering.

**A Bureau of Information :**

We invite inquiries and applications for information and help in providing for that numerous class of sufferers who require special care or house-room which they cannot obtain in their own homes or secure for themselves. **We cannot, however, prescribe, or recommend practitioners.**

**Books for Review :**

Publishers are particularly requested to send advance proofs of any new books of importance whenever possible, as the Editor has made arrangements to publish immediate reviews on a new plan.

**Photographs, Plans, Blocks, and Illustrations :**

It is requested that wherever possible MSS. may be accompanied by illustrations in any of the above forms. The name of the sender and of the article to which it belongs should be written on the back of each photograph, drawing, or block for purposes of identification.

**Local Papers :**

Newspapers containing reports or news paragraphs should **be marked** and addressed to the Sub-Editor.

**The Coupon System :**

A coupon will be found at the bottom of the third inside page of the cover each week. This coupon must be attached to every question or inquiry to which an answer is desired in The Hospital.

### PRACTICAL HELP

FOR THE

**HEALTH VISITOR, DISTRICT NURSE,**

AND

**ALL ENGAGED IN SOCIAL WORK.**

Experience teaches you that your work among the Sick and Suffering and the Poor and Needy would be less harassing and much more effective could you obtain PRACTICAL HELP in the many problems you encounter in the daily round.

Maybe, someone in your Parish or District would benefit by a course of Sanatorium Treatment, another is needing a surgical appliance, or a third, after years of honest toil, is requiring with advancing years whole or partial maintenance.

You know there is provision for these cases, and you would like to assist; your intentions are good, but you are at a loss for want of information.

It is to help you in such difficulties that The Hospital recently inaugurated a Bureau of Information through the medium of which it places at the disposal of its readers, free of all charge, the accumulation of years of experience in Charitable Work.

The Bureau cordially invites enquiries on all subjects, and these are dealt with by those best qualified to answer the questions.

In the interest of your vocation it is essential that you obtain a fuller knowledge of the scope and facilities provided by The Hospital Bureau of Information, and a descriptive booklet will be sent, post free, on application to the Editor, The Hospital Bureau of Information, The Hospital Building, 28 and 29 Southampton Street, Strand, London, W.C.

### Manager's Notices.

**All letters on business matters must be addressed to The Manager, "The Hospital," 28 and 29 Southampton Street, London, W.C.**

**Advertisements :**

To ensure insertion, all advertisements for The Hospital must reach the Manager not later than Wednesday at noon in each week. For scale of charges see pages v and 2.

**Subscriptions,** which may commence at any time, are payable in advance. Cheques and Post-Office Orders should be crossed London County and Westminster Bank, Covent Garden Branch, and made payable to the Manager of The Hospital.

**Remittances :**

**It is especially requested that remittances be made by Cheque or Postal Order, and in halfpenny stamps only when the amount is under sixpence.**

